# Investigating the Association of Wallerian Degeneration and Diaschisis After Ischemic Stroke With BOLD Cerebrovascular Reactivity

**DOI:** 10.3389/fphys.2021.645157

**Published:** 2021-06-24

**Authors:** C. H. B. van Niftrik, M. Sebök, G. Muscas, S. Wegener, A. R. Luft, C. Stippich, L. Regli, J. Fierstra

**Affiliations:** ^1^Department of Neurosurgery, University Hospital Zurich, University of Zurich, Zurich, Switzerland; ^2^Clinical Neuroscience Center, University Hospital Zurich, University of Zurich, Zurich, Switzerland; ^3^Department of Neurosurgery, Careggi University Hospital, University of Florence, Florence, Italy; ^4^Department of Neurology, University Hospital Zurich, University of Zurich, Zurich, Switzerland; ^5^Department of Neuroradiology, University Hospital Zurich, University of Zurich, Zurich, Switzerland

**Keywords:** blood oxygenation-level dependent, functional magnetic resonance imaging (fMRI), Wallerian degeneration, diaschisis, H_2_O–PET, cerebrovascular reactivity

## Abstract

**Introduction:**

Wallerian degeneration and diaschisis are considered separate remote entities following ischemic stroke. They may, however, share common neurophysiological denominators, since they are both related to disruption of fiber tracts and brain atrophy over time. Therefore, with advanced multimodal neuroimaging, we investigate Wallerian degeneration and its association with diaschisis.

**Methods:**

In order to determine different characteristics of Wallerian degeneration, we conducted examinations on seventeen patients with chronic unilateral ischemic stroke and persisting large vessel occlusion, conducting high-resolution anatomical magnetic resonance imaging (MRI) and blood oxygenation-level dependent cerebrovascular reactivity (BOLD-CVR) tests, as well as Diamox ^15^(O)–H_2_O–PET hemodynamic examinations. Wallerian degeneration was determined using a cerebral peduncle asymmetry index (% difference of volume of ipsilateral and contralateral cerebral peduncle) of more than two standard deviations away from the average of age-matched, healthy subjects (Here a cerebral peduncle asymmetry index > 11%). Diaschisis was derived from BOLD-CVR to assess the presence of ipsilateral thalamus diaschisis and/or crossed cerebellar diaschisis.

**Results:**

Wallerian degeneration, found in 8 (47%) subjects, had a strong association with ipsilateral thalamic volume reduction (*r*^2^ = 0.60) and corticospinal-tract involvement of stroke (*p* < 0.001). It was also associated with ipsilateral thalamic diaschisis (*p* = 0.021), No cerebral peduncular hemodynamic differences were found in patients with Wallerian degeneration. In particular, no CBF decrease or BOLD-CVR impairment was found.

**Conclusion:**

We show a strong association between Wallerian degeneration and ipsilateral thalamic diaschisis, indicating a structural pathophysiological relationship.

## Introduction

Ischemic stroke can lead to neurophysiological and structural brain tissue changes, distant from the acute, primary focal lesion. These changes can be seen as post-stroke phenomena, known as Wallerian degeneration and diaschisis (i.e., crossed cerebellar diaschisis and ipsilateral thalamic diaschisis).

Wallerian degeneration is a secondary retrograde degeneration of descending fiber tracts or anterograde *trans*-synaptic degeneration after acute ischemic stroke and is considered a pure structural phenomenon (Zhang et al., 2012). Diaschisis on the other hand, encompasses remote neurophysiological changes, caused by neuronal deactivation, due to a supratentorial lesion (e.g., ischemic stroke). Although both have a similar pathophysiological origin (i.e., disruption of fiber tracts), the main imaging feature of Wallerian degeneration is structural change (i.e., atrophy), whereas diaschisis displays functional–i.e., neurophysiological–changes, such as a local reduction in cerebral blood flow and hypometabolism (Baron et al., 1981; Pantano et al., 1986; Sebök et al., 2018; van Niftrik et al., 2019). In addition, we have recently reported novel hemodynamic and structural imaging aspects related to diaschisis. Both ipsilateral thalamic diaschisis as well as crossed cerebellar diaschisis could be well detected with novel blood oxygenation-level dependent cerebrovascular reactivity (BOLD-CVR) (Sebök et al., 2018; van Niftrik et al., 2019). Ipsilateral thalamic diaschisis exhibited impaired BOLD-CVR in and thalamic atrophy whereas presence of crossed cerebellar diaschisis detected with BOLD-CVR showed an association with hemodynamic impairment, thereby further supporting the concept of a vascular component (Sebök et al., 2021; van Niftrik et al., 2019; von Bieberstein et al., 2020).

From a time course perspective, Wallerian degeneration can be detected as soon as a couple of days in young children (Mazumdar et al., 2003), whereas for adults it is usually first detected 2–3 weeks after the initial stroke. Diaschisis, on the other hand, may be found within hours or even minutes (Kamouchi et al., 2004; Zhang et al., 2012), and may even “disappear” over time (Feeney and Baron, 1986). This may, in some cases, be related to timely blood flow restoration (Sobesky et al., 2005). Interestingly, brain regions, such as the cerebellum and thalamus, where diaschisis persists 2–3 weeks after a stroke also show atrophy, thereby further indicating that both phenomena share a somewhat common pathophysiological pathway related to fibertract disruption (Tien and Ashdown, 1992; van Niftrik et al., 2019). However, the aforementioned local hemodynamic changes in diaschisis have not been studied for Wallerian degeneration.

Therefore, to better understand the pathophysiology of Wallerian degeneration, we investigate novel structural and hemodynamic determinants for Wallerian degeneration and its association with diaschisis. In order to achieve this, we quantified atrophy of the cerebral peduncle on high-resolution T1-weighted images of patients with chronic (>3 weeks) anterior circulation stroke, as compared to an age-matched group of subacute stroke patients (<2 weeks) and a healthy control group. Measuring cerebral peduncular atrophy is a good indicator of Wallerian degeneration because its fibers run in parallel and it is localized remotely from all stroke lesions (Mark et al., 2008; Burke et al., 2014). Hemodynamic features were investigated using the combination of BOLD-CVR and Diamox challenged ^15^O(H_2_O)-Positron Emission Tomography (PET).

## Materials and Methods

### Subjects and Ethical Approval

All data can be made available upon request to the corresponding author (BvN). For this study, we considered data from subjects with chronic (>3 weeks) unilateral symptomatic persisting internal carotid artery or middle cerebral artery occlusion or stenosis, who participated in an ongoing prospective BOLD-CVR–Diamox challenged H_2_O PET comparison study at the University Hospital Zurich. A maximal of 6 weeks was allowed between both measurements. Subjects with any cerebral peduncular, cerebellar or thalamic lesions on initial or follow-up imaging were excluded. Subjects were also excluded when they showcased new neurological symptoms between the BOLD-CVR and Diamox challenged H_2_O PET as well as endovascular and surgical revascularization between both scans. Prior to enrollment of subjects in the prospective database, the research ethics board of the canton Zurich, Switzerland (KEK-ZH-Nr. 2012-0427) approved this study and informed consent was obtained from all subjects.

To determine cerebral peduncular atrophy, we included age-matched, healthy subjects. These subjects were enrolled based on having a negative history of neurological disease and neurological symptoms. Subsequently, age-matched subjects with acute/subacute stroke (onset of first stroke symptoms < 2 weeks) were also included to assess the validity of manually masking the cerebral peduncle. Subjects with stroke onset between 2 and 3 weeks were excluded due to potential overlap.

This resulted in three study groups:

1. Patients with chronic symptomatic steno-occlusive disease (>3 weeks), i.e., chronic group.
2. Patients with acute/subacute symptomatic steno-occlusive disease (<2 weeks), i.e., acute/subacute group.
3. Healthy cohort.

### Experimental Protocol

#### Assessment of Cerebral Peduncular Atrophy

In all patients, a three dimensional (3D) high-resolution T1-weighted Magnetization Prepared Rapid Acquisition Gradient Echo (MP RAGE) image was acquired. Acquisition parameters of the high resolution T1-weighted MP Rage were: voxel size 0.8 × 0.8 × 1.0 mm^3^ with a field of view 230 × 230 × 176 mm and a scan matrix of 288 × 288 × 176, TR/TE/TI 2200/5.14/900 ms, flip angle 8°.

**FIGURE 1:**
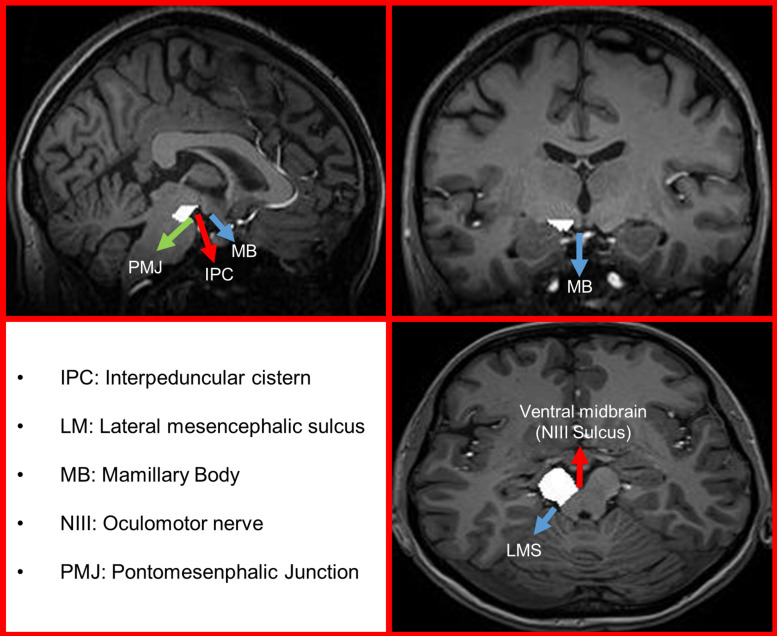
Examplary image of manual masking of a right cerebellar peduncle following Mark et al. (2008).

The left and right cerebral peduncles were manually masked, based on a slight modification of a previously published method (Figure 1) (Mark et al., 2008). Each high-resolution T1-weighted image was separately uploaded in Iplan software (Brainlab, Erlangen, Germany). The medial border of the cerebral peduncle was a straight line drawn through the first point at the corner of the peduncle and the ventral midbrain, and the second point at the lateral sulcus of the midbrain on axial level. The outer margin was then outlined. If any of these structures were not possible to discriminate, the outline on the adjacent layer (either upper or lower) was used as a reference in order to draw the shape. The lower boundary of the cerebral peduncular mask was the ponto-mesencephalic junction, whereas the upper boundary was defined by the mammillary body. The area of the whole cerebral peduncle was then checked in a sagittal plane. These maps were verified by an experienced neuroradiologist, blinded toward the patient groups.

The cerebral peduncular asymmetry index was calculated as follows:

$$\frac{\text{Volume}\,\text{ipsilateral}\,\text{cerebral}\,\text{peduncle}-\text{Volume}\,\text{contralateral}\,\text{cerebral}\,\text{peduncle}}{\text{Volume}\,\text{contralateral}\,\text{cerebral}\,\text{peduncle}}\times 100$$

Wallerian degeneration was deemed present if the volumetric measurements asymmetry index of the cerebral peduncle was more than plus two standard deviations larger than that of the healthy cohort. An index rather than the quantitative value was chosen, to correct for anatomical differences between the subjects.

#### BOLD-CVR and (^15^O)-H_2_O PET Study

MRI: magnetic resonance imaging data were acquired on a 3-tesla Skyra VD13 (Siemens Healthcare, Erlangen, Germany) with a 32-channel head coil. All BOLD fMRI: functional magnetic resonance imaging scans were acquired with the following parameters: axial two-dimensional (2D) single-shot echo-planar imaging sequence planned on the anterior commissure-posterior commissure line plus 20° (on a sagittal image). The voxel size for the BOLD fMRI: functional magnetic resonance imaging scans was 3 × 3 × 3 mm^3^ with an acquisition matrix 64 × 64 × 35 ascending interleaved slice acquisition, slice gap 0.3 mm, GRAPPA factor 2 with 32 ref. lines, repetition time (TR)/echo time (TE) 2,000/30 ms, flip angle 85°, bandwidth 2,368 Hz/Px and a field of view 192 × 192 mm^2^. PET data were acquired on a full ring PET/CT-scanner in 3D mode (PET/CT Discovery STE, GE Healthcare, Chicago, IL, United States). The acquisition parameters and method of processing of the all images is extensively described in previous work (van Niftrik et al., 2017, 2018; Fierstra et al., 2018; Sebök et al., 2018).

To determine BOLD-CVR, we deployed a short, controlled hypercapnic stimulus of ∼10 mmHg above resting CO_2_ levels using the Respiract^TM^ (Slessarev et al., 2007).

Blood oxygenation-level dependent cerebrovascular reactivity, defined as the percentage BOLD signal change per mmHg CO_2_ change, was calculated using a linear regression on a voxel-by-voxel basis between the BOLD signal time course and the CO_2_ time course.

Quantitative BOLD-CVR values were separately determined for the whole brain, ipsilateral supratentorial hemisphere, contralateral supratentorial hemisphere, both thalami, both cerebral peduncles, and both cerebellar hemispheres. For the cerebellum and thalamus, patient individual masks were separately extracted from the subjects’ specific subcortical anatomic parcellation, using Freesurfer software^1^. The technical details of these procedures are described in prior publications (Dale et al., 1999; Fischl et al., 2004). For the healthy cohort, a similar BOLD-CVR analysis was done. Average and standard deviations were calculated, of the cerebellar and thalamic asymmetry indices of the healthy control group (Sebök et al., 2021). Diaschisis (i.e., crossed cerebellar diaschisis and ipsilateral thalamic diaschisis) was deemed present if the asymmetry index in BOLD-CVR was more than plus two standard deviations larger than that of the healthy cohort average (Sebök et al., 2018; van Niftrik et al., 2019).

Concerning H_2_O–PET imaging, due to its qualitative nature, only difference analyses were conducted, which require hemispheric comparison rather than quantitative measurements (Jiang et al., 2010; Fierstra et al., 2018). Supratentorial hemodynamic status was evaluated as the hemispheric difference of the H_2_O PET image before and after Diamox (i.e., PET baseline and PET Diamox, respectively) and PET CVR (percent difference PET baseline and Diamox).

#### Stroke Volume and Location

Using Iplan software (Brainlab, Erlangen, Germany), stroke lesions were manually outlined with Diffusion weighted imaging, T2-Fluid-attentuated-inversion recovery imaging and T1-weighted imaging. In case of multiple stroke lesions, the total stroke volume would embody the sum of all stroke lesions combined. The primary manual masking of the strokes was done using DWI and FLAIR weighted images and then checked on the high-resolution T1 weighted image to correct potential extramarginal masking on lower resolution imaging. Based on the T1-weighted imaging properties, stroke volumes were calculated as the sum of the number of voxels.

Each lesion was also separately analyzed for corticospinal tract involvement. Using Freesurfer free segmentation software (see text footnote 1) (Fischl et al., 2004), the binary masks of the patient individual paracentral lobule and precentral gyrus (gray and white matter) were extracted, and this software was also used to get the individual patient maps for the cerebellum and thalamus. The binary stroke volume map was used as an overlay and corticospinal tract involvement was considered present if the stroke lesion coincided with these regions.

#### Statistical Analysis

We performed the statistical analysis using in-house scripts written in Matlab R2016b (the MathWorks, Inc., Natick, Massachusetts, United States^2^). First, evaluation of normal distribution per variable was done using the Shapiro-Wilk test. Averages of normally distributed, continuous variables from the group exhibiting Wallerian degeneration and the group without Wallerian degeneration were compared by an independent Student’s two tailed *t*-test, where *p* < 0.05 was considered significant. Non-normal distributed, categorical ordinal and dichotomous variables were analyzed with the Mann–Whitney-*U* test. All normally distributed, continuous variables are reported as mean ± standard deviation. Non-normally distributed variables, as well as categorical ordinal variables are presented as median (interquartile range), whereas dichotomous variables are shown as frequency (%, percentage).

To test the relationship of Wallerian degeneration with factors related to long-term volumetric and hemodynamic measurements related to diaschisis, a correlation analysis was done. For normal distributed variables, a Pearson correlation was performed. Other variables were studied using the Spearman Rank-order Correlation. Here, a *p* < 0.05 was deemed significant.

## Results

Seventeen patients with chronic stroke (>3 weeks) met the inclusion criteria and were included for further analysis. Thirteen subjects with acute/subacute stroke and 17 age-matched, healthy subjects were included. The mean age was 58 ± 5 years and 70% of the participants were men. The mean time delay between BOLD-CVR and PET measurements was 16 ± 22 days with a maximum of 38 days. The relevant baseline characteristics of all healthy subjects are shown in Table 1, while all relevant characteristics of all patients with chronic stroke are shown in Table 2. No significant difference between subjects with acute/subacute stroke in baseline parameters and stroke volume was found.

**TABLE 1:** Characteristics of healthy cohort.

	Total cohort (*N* = 17)
Age	59.4 ± 11.1
Sex, *n* Male (%)	12 (66.6)
Smoking *n* (%)	1 (6)
Hypertension *n* (%)	3 (18)
Mean CVR whole brain	0.19 ± 0.04
Mean CVR left cerebral peduncle	0.20 ± 0.08
Mean CVR right cerebral peduncle	0.19 ± 0.08
Volume left cerebral peduncle (cm^3^)	2.20 ± 0.27
Volume right cerebral peduncle (cm^3^)	2.16 ± 0.24
Cerebral peduncle volume AI (%)	1.5 ± 4.5%
CVR Thalamic AI (%)	4.1 ± 8.0%
Volume thalamus left (cm^3^)	0.74 ± 0.09
Volume thalamus right (cm^3^)	0.67 ± 0.06
CVR Cerebellar AI (%)	1.0 ± 5.5%

**TABLE 2:** Relevant clinical and baseline characteristics of patients.

	Total cohort* (*N* = 17)	Wallerian degeneration positive group (*N* = 8)	Wallerian degeneration negative group (*N* = 9)	*p*-value**
Age (mean ± SD)	58.3 ± 12.9	58.1 ± 13.5	58.5 ± 13.2	0.95
Sex, *n* Male (%)	13 (76)	5 (63)	8 (89)	0.37
Smoking *n* (%)	12 (71)	4 (50)	8 (89)	0.09
Hypertension *n* (%)	12 (71)	4 (50)	8 (89)	0.09
Hypercholesterolemia *n* (%)	5 (29)	5 (29)	12 (71)	0.50
Obesity *n* (%)	2 (12%)	3 (38)	2 (22)	0.93
Diabetes *n* (%)	1 (6%)	1 (13%)	1 (11)	0.34
Mean CO_2_ baseline (mmHg)	37.8 ± 2.67	37.4 ± 2.5	38.2 ± 2.9	0.56
Mean CO_2_ hypercapnia (mmHg)	47.2 ± 2.2	46.8 ± 2.5	47.6 ± 2.0	0.48
Mean CO_2_ stepchange (mmHg)	9.4 ± 1.3	9.4 ± 1.2	9.4 ± 1.5	0.98
Mean time after stroke (weeks)	41.6 ± 59	32.3 ± 28.8	50.0 ± 78.5	0.56
Stroke volume	7.86 ± 12.07	10.29 ± 17.4	3.19 ± 4.20	0.25

CO_2_, carbon dioxide; mmHg, millimeter mercury; *N*, number; SD, standard deviation.

*Total cohort describes all included chronic (>3 weeks) anterior circulation stroke patients

**p-Value determined between the group with and without Wallerian degeneration.

### Structural Features of Wallerian Degeneration

**FIGURE 2:**
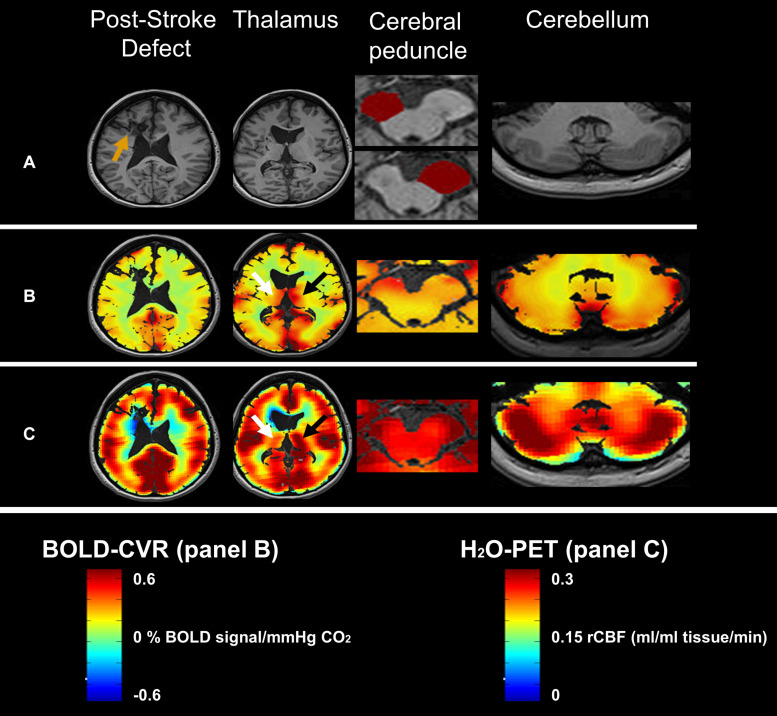
Illustrative image of a 39-year old female patient with Wallerian degeneration (91 weeks post-stroke). Panel **(A)** shows the T1 image of the post-stroke defect (orange arrow), thalamus, left and right cerebral peduncle masks and cerebellum. Note the large difference between the ipsilateral (top: 1.21 cm^3^) and contralateral cerebral peduncle (bottom: 1.65 cm^3^) with a cerebral peduncle asymmetry index of −37%. Panel **(B)** shows the equivalent BOLD-CVR images. Ipsilateral thalamic diaschisis can be seen (white arrow vs black arrow) as a asymmetry in BOLD-CVR (i.e. ipsilateral decrease). No clear BOLD-CVR asymmetry was seen in the cerebral peduncle or cerebellum. Panel **(C)** shows the equivalent 15O-H2O-PET cerebral blood flow images. Here also, thalamic asymmetry can be appreciated without blood flow differences in the cerebral peduncles or cerebellum. Abbreviations: BOLD: blood oxygenation-level dependent, cm: cubic centimeter, min: minute, ml: milliliter, mmHg: millimeter mercury, PET- Positron Emission Tomography, rCBF: relative cerebral blood flow.

Volumetric measurements of the left and right cerebral peduncle in healthy subjects resulted in 2.08 ± 0.29 cm^3^ for the right cerebral peduncle and 2.11 ± 0.33 cm^3^ for the left peduncle. Consequently, the peduncular asymmetry index for healthy subjects was 1.0 ± 5.0%. Wallerian degeneration was then considered present in patients having a peduncular asymmetry index larger than 11% (i.e., exceeding the mean cerebral peduncular asymmetry index of the healthy control group by plus two (2 × 5) standard deviations, see section “Materials and Methods”). In the chronic group, eight (47%) had cerebral peduncular asymmetry indices larger than 11% and were classified as having Wallerian degeneration. In comparison, in the acute/subacute group, none of the cerebral peduncular asymmetry indices reached 11%. An exemplary patient with Wallerian degeneration is depicted in Figure 2.

On average, chronic patients had a markedly lower ipsilateral cerebral peduncular volume in comparison to healthy subjects (1.75 ± 0.33 cm^3^, *p* = 0.003), which was more pronounced in the patients with Wallerian degeneration (1.52 ± 0.26 cm^3^, *p* > 0.001) and not found in the patients without Wallerian degeneration (1.98 ± 0.21 cm^3^, *p* = 0.25). Between patients with and without Wallerian Degeneration, only the volume of the ipsilateral cerebral peduncle differed significantly (*p* = 0.002).

No volumetric differences in the cerebral peduncles were found between healthy subjects, acute/subacute patients and chronic patients without Wallerian degeneration.

Interestingly, stroke volume did not differ between patients with or without Wallerian degeneration; however, as may be expected, the corticospinal tract involvement within the stroke location was seen more often in the group with Wallerian degeneration.

### Hemodynamic Features of Wallerian Degeneration

Mean BOLD-CVR of the healthy cohort for the left and right peduncle was 0.19 ± 0.08, compared to the mean CVR of 0.16 ± 0.09 (*p* = 0.21) for the ipsilateral cerebral peduncle, and a mean of 0.17 ± 0.10 (*p* = 0.41) for the contralateral cerebral peduncle in the chronic stroke patients.

Table 3 shows the hemodynamic features of Wallerian degeneration. Within the cerebral peduncle, BOLD-CVR did not show a difference between chronic stroke patients with Wallerian degeneration and patients without Wallerian degeneration, nor was there a difference in the asymmetry index. Similarly, both the baseline H_2_O–PET and the acetazolamide challenged PET did not show a difference for the cerebellar peduncle.

**TABLE 3:** Structural and hemodynamic findings.

Functional measurement (mean ± standard deviation)	Total cohort (*N* = 17)	Wallerian degeneration positive group (*N* = 8)	Wallerian degeneration negative group (*N* = 9)	*p*-value
Volume ipsilateral cerebral peduncle (cm^3^)	1.75 ± 0.32	1.51 ± 0.27	1.96 ± 0.21	**0.002**
Volume contralateral cerebral peduncle (cm^3^)	1.92 ± 0.32	1.82 ± 0.32	2.01 ± 0.21	0.17
Cerebral peduncle volume AI	−11.0 ± 10.7	−20.33 ± 7.71	−2.71 ± 3.95	
Mean BOLD-CVR ipsilateral cerebral peduncle	0.16 ± 0.09	0.14 ± 0.12	0.17 ± 0.06	0.50
Mean BOLD-CVR contralateral cerebral peduncle	0.17 ± 0.10	0.15 ± 0.12	0.19 ± 0.08	0.41
Cerebral peduncular BOLD-CVR AI (%)	1.09 ± 23.09	−4.53 ± 27.01	7.52 ± 17.45	0.33
Cerebral peduncular PET Baseline AI (%)	6.76 ± 5.49	6.19 ± 4.08	7.4 ± 7.07	0.68
Cerebral peduncular PET Diamox AI (%)	5.32 ± 7.31	6.44 ± 4.76	4.06 ± 9.74	0.55
Ipsilateral thalamic diaschisis*** (%)	9 (47%)	7 (88)	2 (22)	**0.02**
Volume thalamus ipsilateral (cm^3^)	0.57 ± 0.95	0.51 ± 0.07	0.65 ± 0.08	**0.001**
Volume thalamus contralateral (cm^3^)	0.69 ± 0.08	0.67 ± 0.06	0.71 ± 0.10	0.32
Thalamic volume AI (%)	−19.2 ± 16.6	−34.5 ± 24.1	−9.7 ± 12.5	**0.02**
BOLD-CVR ipsilateral thalamus	0.15 ± 0.06	0.12 ± 0.08	0.17 ± 0.05	**0.19**
BOLD-CVR contralateral thalamus	0.18 ± 0.07	0.16 ± 0.09	0.19 ± 0.06	0.62
Thalamic BOLD-CVR AI (%)	20.15 ± 22.98	32.97 ± 29.26	9.36 ± 17.66	0.07
Thalamic H_2_O PET baseline AI (%)	10.65 ± 9.65	14.58 ± 7.82	5.69 ± 10.54	0.10
Thalamic H_2_O PET Diamox AI (%)	10.78 ± 9.35	15.38 ± 8.79	5.08 ± 8.09	0.05
Crossed cerebellar diaschisis*** (%)	7 (36)	4 (50)	3 (33)	0.61
Volume cerebellum ipsilateral (cm^3^)	5.58 ± 0.80	6.06 ± 0.62	5.7 ± 0.92	0.44
Volume cerebellum contralateral (cm^3^)	5.79 ± 0.82	5.88 ± 0.43	5.72 ± 0.10	0.74
Cerebellar volume AI (%)	−1.47 ± 6.80	−2.88 ± 5.19	−0.41 ± 7.99	0.52
Cerebellar BOLD-CVR AI (%)	3.78 ± 18.01	1.82 ± 6.8	−1.90 ± 26.08	0.87
Cerebellar H_2_O PET baseline AI (%)	4.42 ± 5.07	6.11 ± 5.01	2.50 ± 5.62	0.18
Cerebellar H_2_O PET Diamox AI (%)	4.42 ± 3.84	5.45 ± 4.03	2.48 ± 3.44	0.15
Stroke volume (cm^3^)	6.53 ± 12.46	10.29 ± 17.4	3.19 ± 4.20	0.25
Corticospinal tract involvement (%)*	7 (36)	7 (88)	0 (0)	**<0.001**

*AI, asymmetry index; CVR, cerebrovascular reactivity, defined as percentage BOLD signal change per mmHg CO_2_; *N*, number; PET, Positron emission tomography.*

Values are presented as median (interquartile).

*Stroke lesions within the corticospinal tract

***As determined by the asymmetry indices of BOLD-CVR.

### Wallerian Degeneration and Diaschisis

**FIGURE 3:**
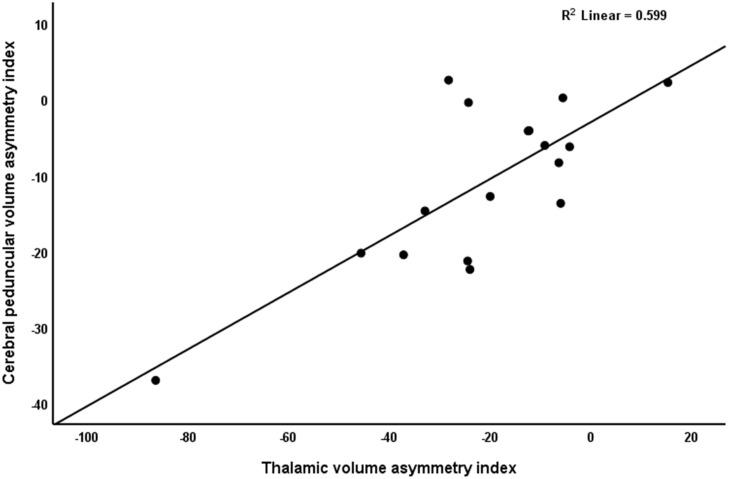
Correlation between cerebral peduncular volume asymmetry index and thalamic volume asymmetry index. Values around zero represent no ipsilateral-contralateral difference. Negative values represent a smaller ipsilateral volume. Note the strong positive relationship between both variables.

Table 3 shows the correlation analysis between Wallerian degeneration and diaschisis. Within the group of chronic patients, ipsilateral thalamic diaschisis was found in nine (53%) patients and crossed cerebellar diaschisis was found in seven (41%). Wallerian degeneration was often found in association with ipsilateral thalamic diaschisis (*p* = 0.021) and so these patients showed a marked increase in neurophysiological and structural variables associated with the thalamus (e.g., an increase in the BOLD-CVR asymmetry index, as well as an increase in both PET asymmetry indices and an decrease in ipsilateral thalamic volume–see Table 3). Moreover, there was a strong positive correlation between the thalamic volume asymmetry index and cerebral peduncular volume asymmetry index (*r* = 0.77, *r*^2^ = 0.59, *p* > 0.001–Figure 3), with more than half of the variance explained (*r*^2^ = 0.60). As for the association between crossed cerebellar diaschisis and Wallerian degeneration, the BOLD-CVR and PET cerebellar asymmetry indices did not differ between both groups, nor was there a correlation to be found between the cerebellar volume asymmetry index and the cerebral peduncular asymmetry index (*r* = −0.03, *p* = 0.93).

## Discussion

### Main Findings

Wallerian degeneration and diaschisis have a common pathophysiological origin (i.e., disruption of fiber tracts). Moreover, persisting diaschisis also causes atrophy, which is the main feature of Wallerian degeneration, thereby perhaps indicating that both are features of a common pathophysiological pathway. However, local hemodynamic changes commonly seen in diaschisis, as well as its association with diaschisis, have not been studied for Wallerian degeneration. In this study, we show that cerebral peduncles showing Wallerian degeneration, do not display hemodynamic features usually found in diaschisis. In particular, no CBF decrease or BOLD-CVR impairment were found. This may be related to the small sample size of this study. We do, however, show a strong association between Wallerian degeneration and ipsilateral thalamic diaschisis, indicating a structural relationship between both pathophysiological entities.

#### The Study of Wallerian Degeneration

In 1849, Augustus Waller observed that following transection of the hypoglossal and glossopharyngeal nerve, the distal portion of the nerve undergoes progressive degeneration (Waller, 1850). Initially found in the peripheral nervous system, where Wallerian degeneration takes only days, the existence of Wallerian degeneration was subsequently discovered in the central nervous system, where it occurred in 2–8 days in young children (Mazumdar et al., 2003) and in adults 2–3 weeks after the initial acute stroke (Warabi et al., 1987; Kuhn et al., 1989; Pujol et al., 1990; Castillo and Mukherji, 1999; Uchino et al., 2004; Vargas and Barres, 2007). Our study found a 47% incidence of Wallerian degeneration in the chronic stroke group, which is similar to the incidence reported by previous publications (Pujol et al., 1990; Uchino et al., 2004). In comparison, none of the subjects within the acute/subacute group showed a large enough cerebral peduncle volume asymmetry to surpass the average from healthy subjects by two standard deviations (i.e., was classified as having Wallerian degeneration), indicating our method to be valid for the masking of the cerebral peduncle.

In this study, the presence of Wallerian degeneration was independent of stroke volume, but showed a strong association with corticospinal tract involvement of the stroke lesion. Moreover, we found a strong association between the presence of Wallerian degeneration and neurological outcome. This has also been found by others, but is in disagreement with some historical papers (Schiemanck et al., 2005, 2006; Mark et al., 2008; Schaechter et al., 2009; Cassidy et al., 2018). In earlier work, infarct volume was correlated with motor outcome, but later studies suggested that corticospinal fiber tract involvement of stroke was a stronger predictor for motor outcome (Saver et al., 1999; Schaechter et al., 2009; Burke et al., 2014; Cassidy et al., 2018). Infarct volume in itself is an inexact variable, which does not say anything about the infarct location (Cassidy et al., 2018). Moreover, the predictive value of stroke lesion volume has more merit in the acute/subacute stroke phase (Stinear et al., 2007; Mark et al., 2008; Cassidy et al., 2018). As Wallerian degeneration is seen in patients with strokes involving the corticospinal fiber tract, it has been hypothesized that the association between Wallerian degeneration and worse neurological improvement was mostly due to the extent of the corticospinal tract involvement (Warabi et al., 1990; Liang et al., 2007, 2008; Lindberg et al., 2007; Puig et al., 2010). The corticospinal tract is the largest fiber tract in the cerebral peduncle and has a direct relationship to motor function. However, determining the integrity of the corticospinal tract supratentorially did not show a strong correlation and Wallerian degeneration was subsequently even identified as an independent measurement of motor impairment and greater disability (Mark et al., 2008; Burke et al., 2014). This might be due to the diversity of the corticospinal tract superior to the cerebral peduncle. The corticospinal tract represents the main motor output pathway and portions arise from different areas like the precentral gyrus, premotor cortex, cingulate motor areas and supplementary motor area (Puig et al., 2017). Only taking the portion descending from the precentral gyrus into account, is an oversimplification of the underlying anatomy. Because all these tracts converge and are first aligned in parallel in the cerebral peduncle, the cerebral peduncle may be the most optimal location to test corticospinal integrity as a whole.

#### The Association With Diaschisis

Diaschisis is a process primarily showing remote neurophysiological changes after a supratentorial lesion (Carrera and Tononi, 2014). Areas with diaschisis experience a decrease in cerebral blood flow, metabolism and BOLD-CVR and can be found directly after the stroke (Baron et al., 1981; De Reuck et al., 1995; Kamouchi et al., 2004; Sebök et al., 2021). These classical hemodynamic features, normally found in diaschisis, were not found in the cerebral peduncle of subjects with Wallerian degeneration. Specifically, no local difference in BOLD-CVR and cerebral blood flow could be detected, indicating a different pathophysiological mechanism for Wallerian degeneration. However, these findings need to be interpreted with caution. Physiologically speaking, a plausible cause for this difference could lie in the fact that white matter fiber tracts are usually much less perfused than other regions, like the thalamus or cerebellum. Smaller changes, in small regions like the cerebral peduncle, would therefore have gone unnoticed. Secondly, the cerebral peduncles are located posterior of the interpeduncular cisterns. It is known that the BOLD signal in regions close to the cerebrospinal fluid can be influenced by artifacts (Thomas et al., 2013). Such artifacts could mask small but significant hemodynamic differences within the cerebral peduncles. This is even more pronounced for H_2_O–PET imaging, as the resolution of those images is significantly lower than that of BOLD-CVR (see Figure 2). Furthermore, despite that both thalami and cerebellum are perfused by the posterior circulation, anatomical variation may account for variability in thalamic perfusion and BOLD-CVR.

The primary feature of Wallerian degeneration is a reduction in volume, due to degeneration of descending fiber tracts or *trans*-synaptic degeneration (Zhang et al., 2012). Diaschisis was thought to be a reversible phenomenon, but atrophy of the area presenting long-term diaschisis has also been found (Pantano et al., 1986; Tien and Ashdown, 1992; van Niftrik et al., 2019). This is more clearly discernable in the thalamus of patients with ipsilateral thalamic diaschisis, than it is discernable in the cerebellum of patients with crossed cerebellar diaschisis (van Niftrik et al., 2019). Our data shows a strong association between thalamic volume decrease and cerebral peduncle volume decrease. Such associations were not found for the cerebellum. This makes it more likely that the majority of ipsilateral thalamic diaschisis is due to disruption of the thalamo-cortico or cortico-thalamic tracts or indirect cerebral peduncular loop fibers. It is less likely to be due to the disruption of the afferent cerebellar-pontine-thalamic tracts. Other tracts, like the frontopontine, temporopontine, parietopontine, and occipitopontine fiber tracts, which are associated with crossed cerebellar diaschisis, also project parallel through the cerebral peduncle (Liu et al., 2007; Kamali et al., 2010). However, it is expected that direct injury of only one of those tracts may results in smaller atrophy–potentially not quantifiable–as discrete Wallerian degeneration of those tracts remains absent. This might explain why the presence of crossed cerebellar diaschisis seems to be independent of Wallerian degeneration.

#### Limitations

In this preliminary study, we have only included 17 subjects with chronic stroke. Therefore, the results should be interpreted with caution. In particular, the small study population most likely resulted in a lack of statistical power in order to sensitively detect hemodynamic differences between Wallerian degeneration and diaschisis. Therefore, a validation study in a larger cohort with correction for other factors should be done. In this study, the cerebral peduncle was manually masked, whereas the cerebellum and thalamus could be masked using patient individual masks obtained from Freesurfer software. This could have resulted in some erroneous measurement. However, as none of the cerebral peduncular asymmetry indices of patients in the acute/subacute stroke group exceeded the threshold of measuring two standard deviations higher than the mean of healthy subjects, we believe the masking was valid. However, optimally, masking should occur without human involvement to get the most unbiased measurements.

## Conclusion

Our data indicate a strong association between Wallerian degeneration and ipsilateral thalamic diaschisis, indicating a structural relationship between both pathophysiological entities.

## Data Availability Statement

The raw data supporting the conclusions of this article will be made available by the authors, without undue reservation.

## Ethics Statement

The studies involving human participants were reviewed and approved by KEK-ZH-Nr. 2012-0427. The patients/participants provided their written informed consent to participate in this study.

## Author Contributions

CN, MS, LR, and JF: study the design. CN, MS, GM, SW, and CS: data acquisition. CN, MS, GM, SW, CS, AL, LR, and JF: data analysis and interpretation and manuscript contribution. All authors contributed to the article and approved the submitted version.

## Conflict of Interest

The authors declare that the research was conducted in the absence of any commercial or financial relationships that could be construed as a potential conflict of interest.

## Footnotes

1 http://surfer.nmr.mgh.harvard.edu

2 http://www.mathworks.com/
